# Multiparametric MRI for local staging in patients with suspected muscle-invasive bladder cancer: study protocol for a multicentre, non-inferiority randomised controlled trial (the BladParadigm study)

**DOI:** 10.1136/bmjopen-2025-100002

**Published:** 2025-08-16

**Authors:** Sebastiaan W van Koeverden, Lisa MC van Hoogstraten, Maarten de Rooij, Marloes van der Leest, Janneke PC Grutters, BladParadigm study group, Arnold Baars, Lambertus ALM Kiemeney, Antoine G van der Heijden

**Affiliations:** 1 Department of Medical Imaging, Radboud University Medical Center, Nijmegen, Gelderland, The Netherlands; 2 Department of Urology, Radboud University Medical Center, Nijmegen, Gelderland, The Netherlands; 3 IQ Health Science Department, Radboud University Medical Center, Nijmegen, Gelderland, The Netherlands; 4 Departments of Urology and Radiology, Radboud University Medical Center, Nijmegen, Gelderland, The Netherlands

**Keywords:** Urological tumours, Magnetic resonance imaging, Bladder disorders, Diagnostic radiology, Quality of Life

## Abstract

**Introduction:**

Muscle-invasive bladder cancer (MIBC) is an aggressive type of cancer. About 50% of patients will die from the disease within 5 years despite radical treatment. This implies that in many patients, the disease has already spread at the time of radical treatment, even though imaging shows no signs of metastasis. We hypothesise that the standard local staging method, transurethral resection of the bladder tumour (TURBT), is partly responsible for tumour cell spread. Furthermore, TURBT (and re-TURBT in many patients) contributes to a significant delay to definitive therapy. The aim of this randomised study is to determine whether multiparametric MRI (mpMRI) of the bladder, in combination with a single outpatient bladder tumour biopsy for histological confirmation, is a safer, faster, less costly and, therefore, more cost-effective diagnostic pathway than TURBT to detect or rule out MIBC.

**Methods and analysis:**

BladParadigm is a two-arm multicentre randomised controlled trial (RCT) conducted in the Netherlands. Over a 3-year period, patients with clinically suspected MIBC without evidence of metastases will be recruited and randomised 1:1 to either TURBT or 3-Tesla mpMRI with same-day outpatient bladder biopsy. The Vesical Imaging Reporting and Data System (VI-RADS) will be used to standardise mpMRI reporting. Patients will undergo definitive treatment based on the results of the TURBT or mpMRI. The study is powered to demonstrate that the mpMRI-based strategy is at least non-inferior to standard TURBT in patients treated with radical cystectomy alone, assuming a relative hazard of 0.55. The required sample size is 360 patients (180 TURBT, 180 mpMRI). The primary outcome is 2-year progression-free survival. Progression will be assessed by imaging, according to the current standard of care. Secondary outcome measures are time to definitive treatment, quality of life (EuroQol 5D-5L), healthcare costs and cost-effectiveness.

**Ethics and dissemination:**

This study has received ethical approval from the Medical Ethical Committee Oost-Nederland (NL83685.091.23). All participants will provide written informed consent prior to inclusion. Findings of this study will be disseminated through peer-reviewed, open-access publications, presentations at scientific conferences and stakeholder briefings.

**Trial registration number:**

NCT05779631.

**STRENGTHS AND LIMITATIONS OF THIS STUDY**

- This is one of the first, large, multicentre, randomised controlled trials comparing a multiparametric MRI (mpMRI)-based diagnostic pathway to current standard of care, both in academic and community hospitals.
- All mpMRI scans follow a standardised Vesical Imaging-Reporting and Data System–based protocol for acquisition and reporting.
- All mpMRI scans will be double-read to ensure robust diagnosis.
- At the end of the study, transurethral resection of the bladder tumour and radical cystectomy specimens of the intervention arm will undergo central review to assess the diagnostic validity of mpMRI.
- Inclusion is based on cystoscopic assessment, which is subjective and may vary between clinicians.

## Introduction

### Background and rationale

 Organ-confined muscle-invasive bladder cancer (MIBC) is one of the very few types of cancer for which the prognosis has not improved for decades. Despite receiving curative treatment, around 50% of patients will die from the disease within 5 years.^1^ This indicates that in many cases, the cancer has already spread at the time of radical treatment, even though imaging techniques show no signs of metastasis.

Transurethral resection of the bladder tumour (TURBT) is the current standard for both pathological confirmation and local staging of bladder cancer, but it has several limitations. Unlike most cancers, which are staged non-invasively through imaging, bladder cancer requires TURBT, an invasive procedure that involves cutting through the tumour and its surrounding vasculature, potentially leading to tumour cell dissemination. Small studies have demonstrated the presence of circulating tumour cells (CTCs) in the blood following TURBT, supporting the hypothesis of procedure-induced tumour cell spread.^2^,^4^ Additionally, TURBT may underestimate muscle invasion due to inadequate sampling and tissue handling, necessitating re-TURBT in up to 30% of cases.^5^,^8^ These limitations may delay definitive treatment and contribute to the persistently poor prognosis of MIBC, despite advances in curative therapies.^9^

In the past decade, multiple studies have shown that multiparametric MRI (mpMRI) can be an effective non-invasive imaging tool for assessing detrusor muscle invasion in patients with bladder cancer. Meta-analyses have reported high sensitivity (83–90%) and specificity (88–90%) for mpMRI.^10^,^12^ A recent UK-based trial (BladderPath)^13 14^ introduced an MRI-directed diagnostic pathway for patients with suspected MIBC and showed that it is feasible to incorporate mpMRI into the diagnostic pathway for patients with bladder cancer, and this led to a significant reduction in time to correct treatment. However, since oncological outcomes such as progression-free survival (PFS) were not evaluated, the BladParadigm trial is designed to evaluate whether an mpMRI-based approach can safely replace TURBT in terms of both effectiveness and costs.

### Objectives

We hypothesise that substituting TURBT with a new diagnostic pathway that includes mpMRI and same-day bladder biopsy for local staging of patients with suspected MIBC will improve the 2-year PFS compared with TURBT. Additionally, we expect that this new pathway will result in a shorter time to definitive treatment, improved quality of life, reduced healthcare costs and that it is cost-effective compared with TURBT.

## Methods and analysis

### Patient involvement

Two representatives from the Dutch patient organisation ‘Bladder or Kidney Cancer’ contributed to the study design, focusing on outcome measures (including quality of life) and procedural feasibility. They serve on the steering committee and will assist in implementing the mpMRI in daily clinical practice if results are favourable. Their feedback was used to adapt the patient information brochure. While patients are not involved in data collection or analysis, their insights guide the dissemination and integration of findings. Study outcomes will be shared via newsletters, magazine articles and with international patient groups.

### Trial design and setting

**Figure 1:**
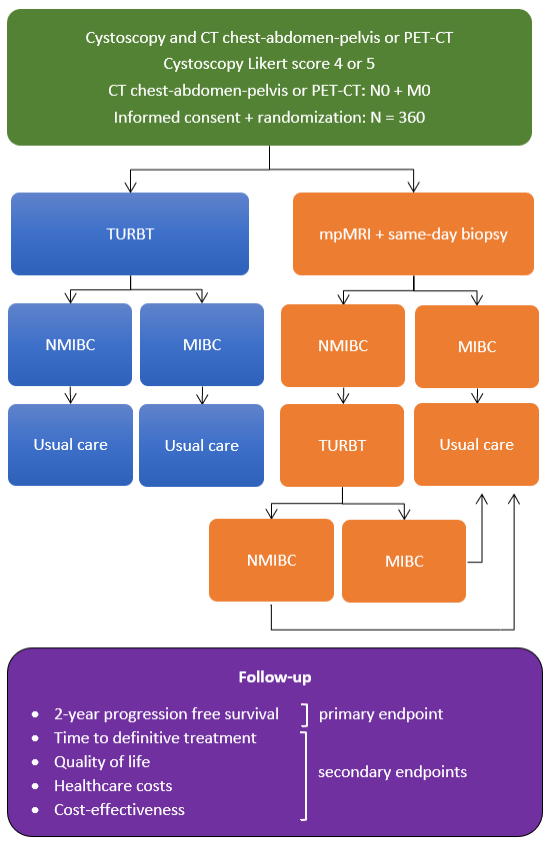
Workflow of the BladParadigm trial. MIBC, muscle-invasive bladder cancer; mpMRI, multiparametric MRI; NMIBC, non-muscle-invasive bladder cancer; PET-CT, positron emission tomography-CT scan; TURBT, transurethral resection of bladder tumour.

Suspicion of MIBC during cystoscopy is scored on a 5-point Likert scale: (1) strongly agree, (2) agree that the lesion is non-MIBC, (3) equivocal, (4) agree or (5) strongly agree that the lesion is MIBC. If the cystoscopy score for suspicion of MIBC is 4 or 5, patients are asked to participate in this study. Patients are randomly assigned (1:1) to either the standard arm (TURBT) or the intervention arm (mpMRI with same-day outpatient biopsy). Randomisation is stratified by study centre and cystoscopy Likert score using alternating block sizes. The planned sample size is 360 patients. The workflow of the trial is shown in figure 1.

### Eligibility criteria

#### Inclusion criteria

- Adults aged ≥18 years.
- Strong suspicion of MIBC during cystoscopy, defined as a Likert score of 4 or 5.
- No evidence of lymph node or distant metastases on CT or positron emission tomography-CT (PET-CT).
- Fit for both diagnostic procedures and definitive treatment with curative intent.

#### Exclusion criteria

- Histologically confirmed non-urothelial bladder tumours.
- History of a solid malignant tumour with less than 5 years’ disease-free survival (except non-melanoma skin cancer or prostate cancer under active surveillance).
- Inability or unwillingness to undergo mpMRI, TURBT or definitive treatment.
- Contraindications for mpMRI (eg, metal in the pelvic area, eGFR (estimated glomerular infiltration rate) ≤30 mL/min).

### Eligibility criteria for sites and professionals

Participating hospitals must treat patients with MIBC. Centres without access to a 3T MRI scanner or without sufficient radiological expertise in bladder MRI will refer eligible patients to one of the other participating centres with the required imaging infrastructure and trained personnel. Radiologists must complete standardised Vesical Imaging-Reporting and Data System (VI-RADS) training prior to participation. Urologists performing TURBT or bladder biopsy must be appropriately qualified and experienced, in accordance with national guidelines and the study protocol.

### Intervention and comparator

#### Standard of care: TURBT

Patients who are randomised to the standard of care arm will undergo TURBT, where both the exophytic part and the tumour base, including detrusor muscle, will be resected according to the European Association of Urology (EAU) guidelines.^15^ Definitive treatment depends on the pathology results, which determine whether or not the tumour is muscle invasive. Patients are then discussed in a multidisciplinary tumour board, following standard practice. For those with NMIBC, the usual care involves TURBT, which may be followed by re-TURBT and/or bladder instillations. In the case of MIBC, definitive treatment typically consists of radical cystectomy (RC) with or without neoadjuvant chemotherapy (NAC) or chemoradiotherapy.

#### Intervention: mpMRI and same-day outpatient biopsy

Patients assigned to the intervention arm will be subjected to an mpMRI and outpatient bladder tumour biopsy the same day. Four academic and five community hospitals will conduct the mpMRI scans. The remaining five hospitals will not conduct the mpMRI themselves due to the unavailability of a 3 Tesla system, anticipated low patient inclusion or limited experience among radiologists. In these cases, the MRIs and the same-day biopsy will be performed at one of the other participating hospitals.

##### Imaging technique

All scans will be conducted on a 3 Tesla scanner from Philips, GE or Siemens. A surface phased array coil will be used. Presence of metal in the pelvic area and an impaired renal function with an individual estimated glomerular filtration rate (eGFR) ≤30 mL/min are considered as absolute contraindications. Relative contraindications, such as allergies, metal presence in other areas than the pelvis and electronic devices, will be left to the discretion of the radiologist. The entire bladder, including internal genitalia in both men and women, will be imaged.

##### Patient preparation

It is essential to achieve optimal bladder distension for accurate assessment, with a target bladder volume of 300 mL. Optimal distension may vary per patient and location of the tumour. The technician or radiologist will visually assess the bladder filling during the examination, looking for a convex bladder shape and sufficient bladder wall distension at the tumour site. Patients are instructed to urinate 1 hour prior to the procedure and then drink 500 mL of water. If the desired distension is not achieved, the scan will be postponed for 15–30 min to allow better filling. Scopolaminebutyl 20 mg intravenous or, if contraindicated, glucagon 1 mg intramuscular, will be administered to reduce bowel movement.

##### Training radiologists

To recognise and stage bladder tumours according to the VI-RADS,^16^ the dedicated mpMRI radiologists from the Department of Medical Imaging at Radboudumc were trained by Professor V. Panebianco and her team at Sapienza University in Rome, Italy. To ensure consistent high-quality imaging and reporting according to VI-RADS across all hospitals participating in the study, all involved radiologists receive training from the dedicated Radboudumc radiologists. Furthermore, all MRI protocols will be adjusted for each hospital and type of MRI scanner to ensure comparable quality.

##### MRI sequences

Scan parameters, adjusted for the type of MRI scanner, will follow the VI-RADS recommended protocol,^16^ as described in table 1.

**Table 1:** Scan parameters for mpMRI of the bladder according to VI-RADS protocol

Sequence	T2 W 3-directions	DWI 2-directions (ax+sag)	DCE
TR (ms)	>5000	>5000	3.8
TE (ms)	119	61	1.2
Flip angle (degree)	90	90	15
FOV (cm)	23	32	27
Matrix	400×256–320	128×128	192×192
Slice thickness (mm)	3	3	1
Slice gap (mm)	0–0,4	0.3–0.4	0
Number of excitations	2–3	4–10	1
B values (s/mm^2^)		0-800-1000 (up to) 2000	

Ax, axial; DCE, dynamic contrast enhancement; DWI, diffusion-weighted imaging; FOV, field of view; mpMRI, multiparametric MRI; Sag, sagittal; TE, time to echo; TR, repetition time; VI-RADS, Vesical Imaging-Reporting and Data System.

##### MRI reporting

VI-RADS provides a systematic method for reporting bladder MRI results, incorporating T2-weighted imaging, diffusion-weighted imaging (DWI) and dynamic contrast enhancement (DCE) to determine the likelihood of muscle invasion. In VI-RADS, the probability of muscle invasion is assessed using a 5-point scale. A VI-RADS 1 score indicates a highly unlikely occurrence of muscle invasion. With a VI-RADS 2 score, muscle invasion is unlikely. With a VI-RADS 3 score, the presence of muscle invasion becomes equivocal. A VI-RADS 4 score indicates that muscle invasion is likely. And a VI-RADS 5 score signifies a very likely invasion of the muscle, extending beyond the bladder.

##### Central radiology review

Double-readings of the mpMRI scans will be performed at the main investigator’s location (Radboudumc) by a dedicated team of radiologists. In case of discrepancies, a third independent reader at the main investigator’s location will be consulted. Each reader will be blinded to the other’s assessments before finalising conclusions on VI-RADS. After reaching consensus, the final result will be reported to the patient’s urologist. In addition, the inter-reader variability among radiologists will be assessed by calculating the intraclass correlation coefficient.

##### Same-day biopsy

Histological confirmation of urothelial carcinoma will be obtained through an outpatient bladder biopsy procedure performed on the same day. A small tumour fragment will be collected using a cold cup biopsy device during cystoscopy.

##### Treatment and follow-up

Results from the mpMRI and biopsy are discussed in a multidisciplinary tumour board, where definitive treatment plans will be determined. Patients with a VI-RADS score of 1 or 2 will be classified as having NMIBC and will typically undergo TURBT, often supplemented by bladder instillations and/or re-TURBT. Those with a VI-RADS score of 3, 4 or 5 will be classified as having MIBC and will undergo definitive treatment, usually involving RC with or without NAC or chemoradiotherapy.

After treatment, all patients, including those in the standard care arm, will be monitored and progression will be assessed by imaging: (PET-)CT every 6 months until the third year, followed by an annual CT scan thereafter. The minimum follow-up duration for all participants in the study is 2 years from the point of randomisation. It is anticipated that patients enrolled earlier in the study can be monitored for a longer duration, potentially to 4.5 years, compared with those who enrol later.

##### Pathology review

All resected specimens from TURBT and cystectomy will be evaluated and reported by a urogenital pathologist, following standard protocols. At the end of the study, all specimens from the intervention arm—those obtained from TURBT (when mpMRI suggests NMIBC) and RC (without NAC, when mpMRI suggests MIBC)—will undergo central review to assess the diagnostic validity of mpMRI.

It is anticipated that mpMRI will classify a maximum of 10% of patients as NMIBC, who will then undergo TURBT. The remaining 90% of patients will be classified as MIBC, with approximately two-thirds of them treated with RC alone. As a result, a central pathology review will be conducted for approximately n=120 cases. As the central pathology review is performed at the end of the trial, results do not affect the choice of definitive treatment.

### Outcomes

#### Primary outcome

The primary outcome is progression-free survival (PFS) at 2 years after diagnosis. PFS is defined as the time between randomisation and the occurrence of distant metastases, nodal recurrence or death from any cause, whichever occurs first. Patients without evidence of progression will be censored at the last date of assessment. In addition to PFS, time to progression (TTP), defined as the time between randomisation and diagnosis of distant metastases or loco-regional nodal recurrence, will be analysed as well. For TTP, we consider death from any cause as a competing risk.

#### Secondary outcomes

Secondary outcome measures are time until definitive treatment, quality of life, hospital-related healthcare costs and cost-effectiveness.

##### Time to definitive treatment

Time until definitive treatment is defined as the period between randomisation and TURBT for NMIBC and RC with or without NAC, or chemoradiotherapy for MIBC.

##### Quality of life

To assess generic health-related quality of life, the EuroQol 5 Dimension 5 Level (EQ-5D-5L) questionnaire^17^ will be administered to all participating patients on paper at baseline (inclusion in study), 6 months, 12 months and 24 months post-randomisation.

##### Hospital costs

Hospital registries will be used to identify all items, procedures and activities related to bladder cancer management to estimate the associated costs. Unit costs will be based on the guidelines outlined in the Dutch manual for costing studies.^18^ To obtain total costs per patient, resource use will be multiplied by the unit costs.

##### Cost-effectiveness

Cost-effectiveness will be analysed from a healthcare perspective, using a 2-year time horizon. Effectiveness will be measured in terms of quality-adjusted life years (QALYs), following the Dutch guideline for economic evaluation.^19^ QALYs will be based on the patient-reported EQ-5D-5L scores. From these scores, utility values will be calculated using the Dutch tariff.^20^ Based on these utility values, QALYs will be computed per patient using the area under the curve approach. Costs and QALYs in the second year of follow-up will be discounted at a rate of 3% and 1.5%, respectively, based on the Dutch guideline for economic evaluation. Absolute means and mean differences between the arms in terms of costs and QALYs will be calculated. As cost data are generally highly skewed, bootstrapping will be performed to estimate 95% CIs around the costs and effects.^21^ If the new strategy is both more costly and more effective, incremental cost-effectiveness ratios (ICERs) will be calculated by dividing the estimated differences in costs by the difference in QALYs. This will result in incremental costs per QALY gained. If the new strategy is less costly and more effective, it is considered to dominate the standard strategy. If it is less costly and less effective, ICERs will be calculated to derive the additional savings per QALY lost. If the new strategy is more costly and less effective, it is considered to be dominated by the current strategy. Cost-effectiveness acceptability curves will be derived, illustrating the probability of cost-effectiveness against different cost-effectiveness thresholds.^22^ All analyses will be performed on an intention-to-treat basis. The impact of missing data will be assessed with single imputation nested in the bootstrap percentile method.^23^

### Data collection methods

Data will be collected using standardised procedures to ensure consistency and accuracy across all participating centres. Clinical and imaging data, including mpMRI results and biopsy findings, will be recorded in the electronic data capture system CASTOR. Quality of life questionnaires (EQ-5D-5L) will be administered on paper, collected via mail and entered into CASTOR by trained study personnel. All data collection forms and procedures will be pilot-tested prior to study initiation to minimise missing data and measurement errors. Data collectors and outcome assessors will be trained and monitored regularly to maintain data quality.

### Harms

Adverse events (AEs) related to the study procedures will be recorded, but formal AE reporting is waived since both mpMRI with biopsy and TURBT are established, routine clinical procedures with well-known safety profiles. Serious adverse events (SAEs) that are unexpected and related to the study will be reported promptly to the accredited Medical Ethics Committee, except for SAEs expected as part of standard care (eg, haematuria following TURBT). All AEs will be monitored until resolution or stabilisation. A Data Safety Monitoring Board (DSMB) has been appointed to oversee patient safety throughout the trial. No interim safety analyses or early stopping rules are planned due to the low risk of harm. The sponsor will suspend the study and notify the ethics committee immediately if significant safety concerns arise.

### Participant timeline

**Figure 2:**
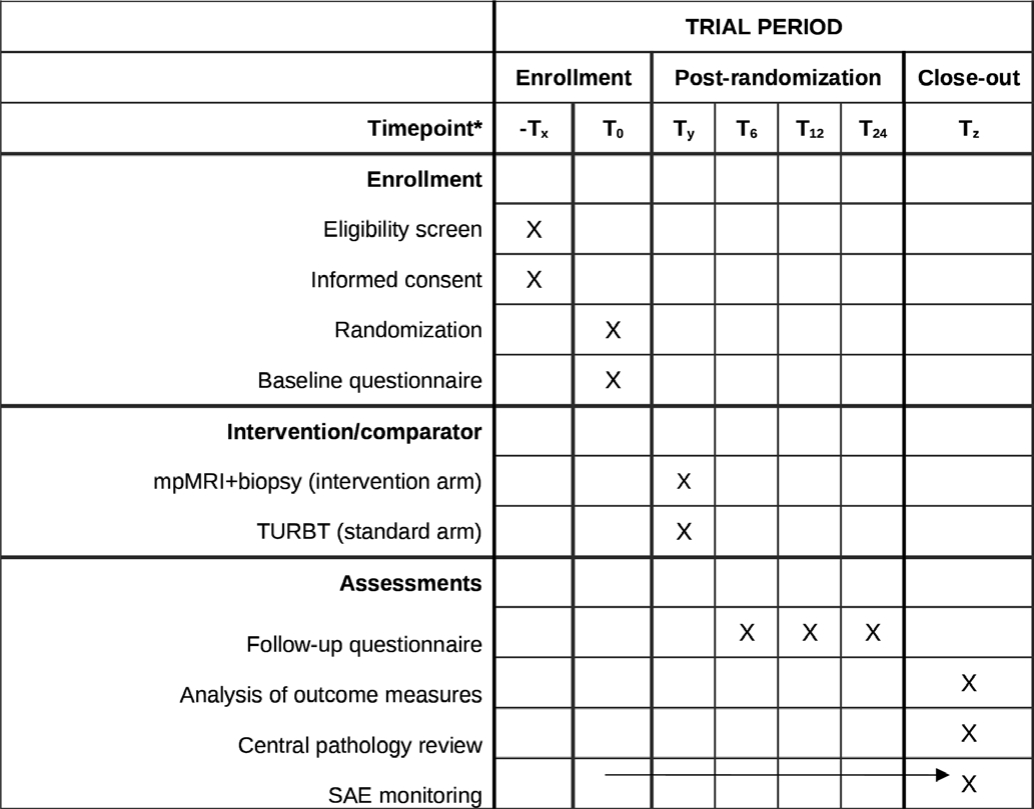
Diagram of the schedule of enrolment, interventions and assessments. *Tx: moment of eligibility screen and signing informed consent; T0: randomisation and baseline questionnaire; Ty: mpMRI+biopsy (intervention arm) or TURBT (standard arm); T6: follow-up questionnaire at 6 months; T12: follow-up questionnaire at 12 months; T24: follow-up questionnaire at 24 months; Tz: close-out, including analyses of outcome measures, central pathology review. SAE monitoring is performed continuously throughout the study. Patients are not actively involved in the close-out. mpMRI, multiparametric MRI; SAE, serious adverse events; TURBT, transurethral resection of the bladder tumour.

The study timeline is as follows (see figure 2): month 1–6: to obtain formal approval of ethics committee and participating centres; month 7–42: patient recruitment, randomisation and intervention; months 7–66 follow-up period (at least 2 years); month 7–66: data collection including data on costs and EuroQol 5D-5L; month 67–72: data analysis and reporting. The study started in July 2023 and is expected to be completed at the end of 2029, including follow-up and final analyses.

### Sample size

The power calculation is based on an intention-to-treat (ITT) time-to-event analysis comparing PFS between the randomisation groups. The following parameters were used: alpha two-tailed=0.05, beta (1-power)=0.20 and 1:1 randomisation. Assuming a relative hazard of 0.55, 88 events are needed. With a baseline event rate of 0.23 per year in the TURBT group, a censoring rate of 0.1 per year, and an average follow-up period of 3.25 years, 117 participants are needed in each group. Despite the fact that adding NAC to RC only provides a 5–8% increase in overall survival after 5 years compared with RC alone, the presence of NAC (or chemoradiotherapy) might still alter the impact of TURBT on prognosis. Therefore, we will conduct a stratified analysis on RC alone vs other (systemic) treatment with curative intent. In the Netherlands, less than 15% of patients with cT2-4aN0M0 MIBC are treated with chemoradiation, a little over 20% with NAC and ~65% with RC alone. That means that 117/0.65=180 patients are needed in each arm. This sample size will provide us with the necessary power to analyse our primary research question in RC-alone patients effectively.

### Recruitment

Patients will be recruited from urology departments of participating hospitals. Eligibility will be assessed based on cystoscopic findings suspicious for MIBC, in the absence of lymph node or distant metastases with imaging. The participating urologists will inform eligible patients about the study and obtain written informed consent. A model of the Dutch informed consent form is provided in online supplemental file 1. We anticipate that the collaboration with 15 hospitals, including academic and large teaching hospitals, will provide a sufficient number of eligible patients to reach the required sample size.

### Randomisation: sequence generation, allocation concealment mechanism and implementation

Patients will be randomised in a 1:1 ratio to either the standard TURBT arm or the intervention arm (mpMRI with same-day biopsy). Randomisation will be performed using a computer-generated allocation sequence, stratified by study centre and by cystoscopy Likert score 4–5 vs <4. A central web-based randomisation system will be used to ensure allocation concealment. The allocation sequence will be generated by an independent data manager not involved in patient enrolment or treatment assignment.

Treating physicians and study staff responsible for patient enrolment will not have access to the allocation sequence. After eligibility is confirmed and informed consent is obtained, the treating urologist or study coordinator will log into the secure system to randomise the patient. The allocated arm will then be revealed to the study team for further planning of diagnostic procedures and treatment.

### Blinding

Given the nature of the interventions, blinding of participants and treating physicians is not feasible. However, the central review of mpMRI scans is performed by blinded radiologists, who will be unaware of clinical information, pathology, and the other readers' scores. Similarly, central pathology review will be performed blinded to the assigned intervention. Data analysts will also be blinded to treatment allocation during statistical analysis. There are no procedures for unblinding, as treatment allocation is not concealed during clinical care.

### Data management

All study data related to data collection, randomisation and trial management will be handled digitally through the CASTOR electronic data capture system. Physical informed consent forms and quality of life (EQ-5D-5L) questionnaires will be collected and stored centrally in a secure location. Both the digital data in CASTOR and the physical documents will be managed and retained in accordance with applicable privacy and security regulations, with a minimum retention period of 15 years. Data management procedures include measures to ensure data quality and integrity, such as double data entry and validation checks to minimise errors. The dataset generated during the study will be made available on reasonable request in compliance with FAIR Data Principles.

### Statistical methods

PFS (and TTP) in both trial arms will be compared in an ITT analysis using Kaplan-Meier curves and a log-rank test. In addition, we will use a proportional hazards regression model with the stratification factors (study centre and cystoscopy score 4/5) as covariates. In case of different distributions of other prognostic factors between both arms, despite the randomisation, we will adjust for these factors as well. Analyses will also be stratified by NAC (yes/no) and the Likert scale score following from visual inspection of the bladder during cystoscopy (score 4–5). We will not conduct any interim analyses.

We will compare the median time to definitive treatment between the two strategies using Kaplan-Meier analysis with a log-rank test. Missing data will be handled using multiple imputation with sensitivity analyses comparing imputed and complete case results. Prespecified subgroup and sensitivity analyses, for instance, stratified by use of neoadjuvant chemotherapy, will be conducted.

### Data monitoring committee

A DSMB has been established to oversee patient safety during the trial. The DSMB is composed of independent experts with relevant expertise in radiology, epidemiology and urology. The DSMB provides independent advice to the trial sponsor regarding safety and trial conduct. Any recommendations made by the DSMB are communicated exclusively to the sponsor. If the sponsor decides not to fully implement the DSMB’s advice, the sponsor will notify the reviewing Medical Ethics Committee (METC) with a detailed explanation.

No interim analyses are planned, and no predefined early stopping criteria have been established for this trial. This decision is based on the fact that both trial arms consist solely of routine clinical procedures, which pose minimal additional risk to participants.

### Trial monitoring

The study sponsor will appoint an independent monitor responsible for overseeing trial conduct and data integrity. Monitoring activities will include centralised and risk-based approaches, supplemented by on-site visits as needed. The monitor will review trial documentation, source data verification and compliance with the protocol and regulatory requirements. Monitoring frequency and scope are defined in a separate monitoring plan. The monitoring process is independent of the investigators and sponsor team. Any findings or recommendations will be communicated to the sponsor and study management to ensure participant safety and data quality.

## Discussion

Currently, TURBT is the cornerstone for local staging of bladder cancer. With TURBT, tumour integrity is violated by cutting through the tumour and removing the tumour in parts. Moreover, during TURBT, overpressure is created for bladder distension which may push cancer cells into the circulation. Some other small studies^2^,^4^ support the hypothesis of distant tumour seeding through this technique, as CTCs have been found in blood samples after TURBT in patients with MIBC where CTCs were not detected prior to the procedure. The presence of CTCs before definitive treatment has been linked to a higher risk of progression.^24^ Consequently, TURBT may contribute to poorer survival rates in MIBC. This is shown in the study of Huang *et al*, where a higher risk of metastases and a lower PFS was found in patients who underwent diagnostic TURBT compared with patients who underwent cystoscopy with biopsy alone before radical cystectomy.^25^

MpMRI, on the other hand, is non-invasive, has minimal complications, requires no hospitalisation and can potentially be performed more quickly and at a lower cost. Numerous studies over the past decades have shown that mpMRI is an excellent tool for distinguishing muscle invasion; various meta-analyses report excellent sensitivity (83–90%) and specificity (88–90%).^10^,^12^ However, this has not yet led to a widespread change in the approach to determine whether a bladder tumour is muscle invasive or not. Possible causes of this are outdated guidelines, local practices, financial considerations and limited knowledge or training among radiologists and urologists.

Previous studies focus on the question of whether mpMRI is capable of differentiating between a muscle-invasive and non-muscle invasive tumour. In our study, we focus on 2-year PFS and, additionally, time until radical treatment, quality of life, hospital-related healthcare costs and cost-effectiveness of an mpMRI-based diagnostic approach and compare this to the current standard of care TURBT.

To the best of our knowledge, the BladderPath study is the only other trial that is investigating an mpMRI-based diagnostic pathway for bladder cancer staging. Results on feasibility and time to correct therapy were recently published.^13 14^ The introduction of an MRI-based diagnostic approach led to a 45-day reduction in time to correct therapy. The findings from this study emphasise the importance of staging patients suspected of MIBC with mpMRI rather than TURBT. However, survival data were not evaluated due to insufficient enrolment due to the COVID-19 pandemic. To optimise our study design, we have consulted with the principal investigator of the BladderPath trial and made several adaptations, including the use of a 3T mpMRI (instead of a 1.5T mpMRI as done in the UK trial), evaluating PFS at 2 years after randomisation as our primary endpoint, and a more stringent selection of only patients with a suspicion of MIBC (Likert score 4 or 5 during cystoscopy, instead of all patients with gross haematuria). The latter, however, could also be considered a limitation, as cystoscopic-based assessment of potential muscle invasion is subject to variability and potential inaccuracies. This was shown in two studies on accuracy of cystoscopy where the prevalence of MIBC among patients with a Likert score of 4 or 5 was 72% and 78%, respectively.^26 27^

Equal to the BladderPath trial, follow-up will be at least 2 years for all patients. Given that most patients who experience progression will have progression in the first 2 years after radical treatment, 2 years is a sufficiently large time window for follow-up. It would be of added value to extend follow-up in this trial beyond 2 years to evaluate long-term results, but due to feasibility constraints, this was not possible. Patients will, however, be monitored long-term according to standard of care.

Another methodological consideration is the use of VI-RADS 3 as a threshold for classifying patients as MIBC. In the original VI-RADS paper, a score of 3 is considered equivocal.^16^ While more recent studies report high diagnostic performance for VI-RADS≥3—with sensitivities of 87–91% and specificities of 82–97%^12 28 29^—the risk of overtreatment in non–muscle-invasive cases cannot be entirely excluded. A meta-analysis by Woo *et al* (n=1170) found a sensitivity and specificity of 92% and 84% for VI-RADS 3–5, compared with 72% and 96% for VI-RADS 4–5, respectively. Although a higher cut-off would reduce false positives, we chose to include VI-RADS 3 in the MIBC category for two reasons. First, the pre-test probability of MIBC in our study population is high (72–78%) because patients are only included if cystoscopy suggests a high likelihood of muscle invasion (Likert score 4 or 5). In this context, the positive predictive value of VI-RADS 3–5 rises to 93.6–95.4%. Second, although inter-reader variability in mpMRI interpretation remains an inherent limitation, the use of central double reading with consensus reporting is expected to improve consistency and reduce the number of equivocal (VI-RADS 3) classifications.

We acknowledge that overstaging may lead to overtreatment, particularly in patients with large, aggressive-looking tumours that may still be non-muscle-invasive. In clinical practice, however, such tumours are often treated more aggressively regardless of histological confirmation, and radical cystectomy is not uncommon in these cases. Although there is no direct evidence quantifying overtreatment in this context, we estimate that its occurrence is low—likely below 5%—corresponding to a positive predictive value above 95%. Conversely, if mpMRI understages an MIBC case and incorrectly classifies it as NMIBC, the patient will still undergo TURBT as per protocol, allowing for detection of muscle invasion at that point. In such cases, the primary consequence could be a delay of several days due to the initial mpMRI and biopsy procedure.

Notwithstanding these limitations, if the results of this RCT show that this new diagnostic approach combining mpMRI with a same-day bladder biopsy is a reliable, safer, faster and more economical alternative for local staging of bladder cancer compared with TURBT, it will pave the way for a paradigm shift in bladder cancer staging.

### Ethics and dissemination

This study (registration number: NL83685.091.23) has received ethical approval from the Medical Ethical Committee Oost-Nederland. All participants will provide written informed consent prior to inclusion.

### Dissemination policy

Findings of this study will be disseminated through open-access, peer-reviewed publications, presentations at scientific conferences and stakeholder briefings.

### Protocol amendments

Amendments to the protocol will be submitted to and must be approved by the METC before implementation. All participating centres and investigators will be informed of approved changes to ensure compliance and maintain study integrity.

### Consent or assent

All participants will provide written informed consent before any study procedures are performed. The consent process will be conducted by trained research staff who will ensure that participants understand the study purpose, procedures, risks and benefits. For participants unable to provide consent, assent procedures will follow applicable regulations.

### Confidentiality

All participant information obtained during the trial will be handled confidentially and stored securely in compliance with applicable data protection regulations. Personal identifiers will be replaced with unique study codes to ensure participant anonymity. Access to identifiable data is restricted to authorised study personnel only. Data will be stored in secure systems (eg, Castor) and physical documents (eg, questionnaires) will be kept in locked archives. Data sharing outside the study team will occur only in anonymised form and in accordance with legal and ethical guidelines.

### Ancillary and post-trial care

The sponsor has liability insurance in accordance with Dutch legal requirements that covers damage to research participants caused by the study, including injury or death. This insurance applies to any damage that becomes apparent during the study or within 4 years after its completion. Participants will receive usual care and follow-up according to standard clinical practice after the trial. No additional ancillary or post-trial care beyond standard treatment is planned.

### Trial registration

ClinicalTrials.gov identifier: NCT05779631. Registered on 22 March 2023.

### Protocol and statistical analysis plan

Both the full study protocol and the statistical analysis plan are available on reasonable request from the corresponding author. Additionally, the study protocol will be published in a peer-reviewed journal.

## Supplementary material

10.1136/bmjopen-2025-100002 online supplemental file 1
