# Supplementary material for: Multiparametric MRI for local staging in patients with suspected muscle-invasive bladder cancer: study protocol for a multicentre, non-inferiority randomised controlled trial (the BladParadigm study)
Source: BMJ Open. 2025 Aug 16;15(8):e100002. doi: 10.1136/bmjopen-2025-100002 (PMC12359491; doi:10.1136/bmjopen-2025-100002)
Supplement: online supplemental file 1 [file bmjopen-15-8-s001.docx]

**PATIËNTEN-INFORMATIEFORMULIER**

**‘BLADPARADIGM’ STUDIE**

**Multiparametrische MRI bij patiënten met verdenking op spier-invasieve blaaskanker: een nieuwe methode om het stadium van de tumor vast te stellen**

Geachte heer/mevrouw,

U krijgt deze brief omdat bij u een blaastumor is vastgesteld die waarschijnlijk in de spierlaag van de blaas is gegroeid (spier-invasieve blaastumor).

Op dit moment vindt een groot onderzoek plaats in Nederland naar de beste methode om deze ingroei in de spier vast te stellen: de zogenaamde ‘BladParadigm’ of ‘Blaaskanker Paradigma’ studie. Met deze informatiebrief willen we u vragen of u aan dat onderzoek mee wilt doen. U leest hier om wat voor onderzoek het gaat, wat het voor u betekent, en wat de voordelen en nadelen zijn van meedoen aan de studie.

1. **Wie voert dit onderzoek uit?**

Dit onderzoek wordt uitgevoerd door de afdeling Urologie van het Radboud universitair medisch centrum (Radboudumc) in Nijmegen, samen met een groot aantal urologie- en radiologie-collega’s in verschillende ziekenhuizen in Nederland.

Ook de ziekenhuizen die op het terrein van blaaskanker nauw samenwerken met de UMC’s, zoals uw ziekenhuis, doen mee. De projectleiders van het onderzoek zijn Dr. Toine van der Heijden, oncologisch uroloog, en Prof. Bart Kiemeney, epidemioloog, van het Radboudumc. Voor het onderzoek zijn 360 deelnemers nodig en het duurt 6 jaar. Er is financiering voor het onderzoek ontvangen van de Nederlandse organisatie voor gezondheidsonderzoek en zorginnovatie (ZonMW). De medisch-ethische toetsingscommissie Oost-Nederland heeft het onderzoek goedgekeurd.

1. **Wat is de achtergrond van het onderzoek?**

Blaaskanker is een veelvoorkomende ziekte. Elk jaar krijgen ongeveer 7000 mensen in Nederland blaaskanker. Voor de meeste van die patiënten is een relatief eenvoudige behandeling voldoende en de overleving van deze patiënten is daarna zeer goed.

Maar ongeveer 1600 patiënten hebben een tumor die in de spierlaag van de blaas is gegroeid (spier-invasieve blaastumor). Bij die patiënten is een zeer ingrijpende behandeling nodig. De kans op verergering van de ziekte (dat noemen we progressie) en overlijden is afhankelijk van de uitgebreidheid (ofwel het stadium) van de ziekte. Voor de keuze van behandeling (eenvoudig of ingrijpend) is het dus belangrijk om zeker te weten of er echt sprake is van ingroei van de tumor in de blaasspier. Om dit goed te kunnen beoordelen wordt de blaastumor eerst geheel of gedeeltelijk verwijderd door middel van een kijkoperatie via de plasbuis. Er zijn echter aanwijzingen dat deze standaard kijkoperatie ook risico’s met zich meebrengt.

Bij de meeste vormen van kanker zijn CT en/of MRI scans en het onderzoeken van een klein stukje tumor (biopsie) voldoende om de juiste behandeling te kiezen. Maar bij blaaskanker is het al tientallen jaren voorgeschreven om de tumor zo ver mogelijk te verwijderen voordat de definitieve behandeling plaatsvindt. Dat gebeurt dus met een kijkoperatie via de plasbuis, waarbij de tumor stukje voor stukje wordt weggesneden. Volgens enkele zeer kleine onderzoeken die sinds 2015 zijn uitgevoerd komen bij sommige patiënten door deze kijkoperatie tumorcellen in de bloedbaan terecht. De meeste van deze cellen gaan al snel dood en kunnen daarom geen kwaad. Maar in theorie kunnen deze tumorcellen ook zorgen voor verspreiding van de ziekte in het lichaam. Dat noemen we uitzaaiing. Of dit echt zo is, is nog niet aangetoond.

De laatste jaren hebben meerdere onderzoeken laten zien dat het ook mogelijk is om met een MRI scan van de blaas aan te tonen dat een blaastumor in de spierlaag is gegroeid. Een MRI scan, samen met het nemen van een biopt uit de tumor, zou dus een alternatief kunnen zijn voor de kijkoperatie die iedereen nu krijgt.

1. **Wat is het doel van het onderzoek?**

In deze studie wordt onderzocht of een MRI scan van de blaas in combinatie met het nemen van een biopt van de tumor veiliger is voor patiënten en betere uitkomsten geeft dan het geheel of gedeeltelijk verwijderen van de tumor door middel van een kijkoperatie. Tegelijkertijd willen we kijken of met de MRI scan de tijd tot de definitieve behandeling (dat is meestal het verwijderen van de gehele blaas na een chemotherapie-behandeling of een combinatie van chemotherapie en radiotherapie) korter is, omdat met een MRI scan de wachttijd voor de kijkoperatie wordt vermeden. Uit dit onderzoek kan zo blijken of de diagnostiek van deze vorm van blaaskanker kan veranderen en dus verbeteren.

1. **Hoe verloopt het onderzoek?**

Uw uroloog heeft bij u in de blaas gekeken en geconcludeerd dat er mogelijk ingroei van de tumor is in de spierlaag van de blaas. Ook is er bij u al een CT scan uitgevoerd waarbij geen uitzaaiingen van de blaaskanker zijn gezien. Normaal gesproken zou de eerstvolgende stap zijn om een kijkoperatie via de plasbuis te doen. Maar nu vragen wij u om mee te doen aan dit onderzoek.

Wanneer u aangeeft te willen deelnemen aan het onderzoek zal door middel van loting worden vastgesteld in welke van twee groepen u wordt ingedeeld. De loting gebeurt met een computer zonder dat uzelf of uw behandelend arts daar invloed op kan uitoefenen. De helft van de patiënten zal loten voor het alternatief voor de kijkoperatie. Deze patiënten krijgen een MRI scan van de blaas en daarna wordt op dezelfde dag een klein stukje van de tumor (zogenaamd biopt) weggenomen tijdens blaasspiegeling (cystoscopie). Dit is hetzelfde onderzoek op de polikliniek als u al eerder heeft gehad. De andere helft van de patiënten zal loten voor de standaard behandeling: de kijkoperatie, waarbij de tumor geheel of gedeeltelijk wordt verwijderd.

Nadat u de kijkoperatie heeft gehad dan wel de MRI scan met het biopt zal alles verder weer standaard verlopen. Uw behandelend arts zal samen met u bespreken uit welke definitieve behandelingen u kunt kiezen. Wij zullen u daarna 2 jaar lang volgen in het kader van het onderzoek om te kijken hoe het met u gaat.

In onderstaande figuur ziet u een schema van de studie.


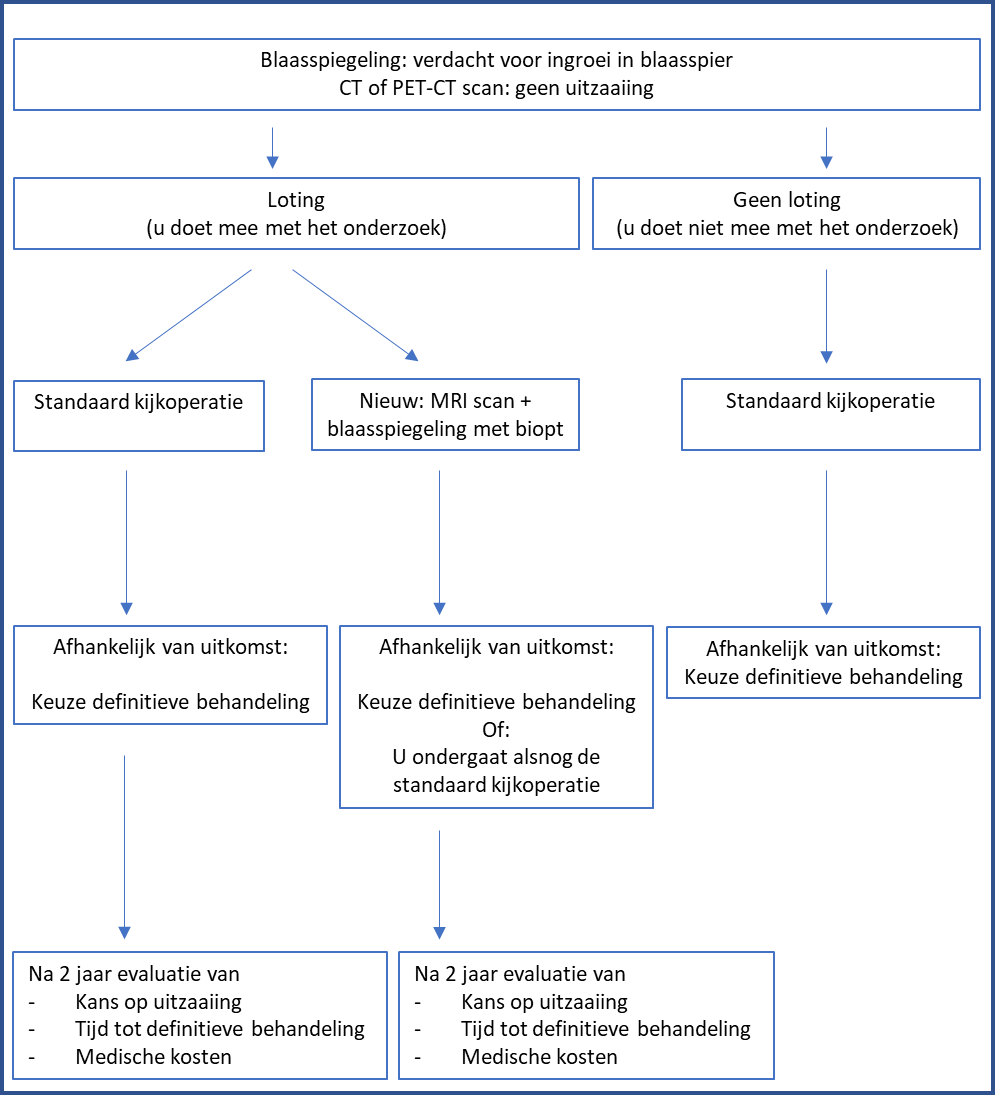


1. **Wanneer kunt u wel of niet meedoen aan het onderzoek?**

U kunt meedoen aan het onderzoek als:

- De uroloog bij u een blaastumor heeft vastgesteld die waarschijnlijk in de spierlaag van de blaas is gegroeid
- Er een CT van de borst en buik is gemaakt waarop geen uitzaaiingen te zien zijn
- U 18 jaar of ouder bent en op het toestemmingsformulier heeft aangegeven mee te willen doen met deze studie

U kunt niet meedoen aan de studie als:

- U niet fit genoeg bent om een kijkoperatie te ondergaan
- U geen MRI scan wilt of kunt ondergaan
- U al eerder kanker heeft gehad, met uitzondering van enkele vrij onschuldige vormen van huidkanker of prostaatkanker, of als dit langer dan 5 jaar geleden is.

1. **Wat is anders dan de normale zorg?**

Als u inloot in de groep die een MRI scan krijgt, krijgt u naast die MRI scan ook een blaasspiegeling extra waarbij een stukje uit de tumor wordt afgenomen. Dat stukje tumor wordt door de patholoog onderzocht om vast te stellen of en om wat voor type blaaskanker het gaat. De blaasspiegeling en MRI vinden plaats tijdens een poliklinisch bezoek. U hoeft er niet voor opgenomen te worden. De standaard kijkoperatie hoeft u dus niet te ondergaan.

Als u inloot voor de standaard zorg krijgt u wel die kijkoperatie waarbij de tumor geheel of gedeeltelijk wordt verwijderd. U verblijft dan meestal 1 of soms enkele nachten in het ziekenhuis. Ook zullen wij u, verspreid over 2 jaar, maximaal 4 keer vragen om een vragenlijst in te vullen om meer inzicht te krijgen in hoe het met u gaat. Het invullen van de vragenlijst kost per keer maximaal 5 minuten.

1. **Wat zijn mogelijke voor- en nadelen van deelname aan dit onderzoek?**

Als u inloot in de standaard-praktijk groep dan ondergaat u de kijkoperatie waarbij de tumor in kleine stukjes zal worden verwijderd. Geen enkele methode om het stadium van de ziekte vast te stellen is 100% nauwkeurig. We weten dat bij een kijkoperatie bij 30% van de patiënten ten onrechte wordt gedacht dat de tumor niet in de spier groeit. Daarom worden patiënten vaak onderbehandeld en wordt hun definitieve behandeling ten onrechte uitgesteld.

De kijkoperatie is een vaak uitgevoerde procedure en de bijwerkingen zijn bekend, bijvoorbeeld pijn en ongemak, bloed bij de urine en problemen met plassen. Na de procedure moet u een of enkele dagen in het ziekenhuis blijven om te herstellen. Het duurt zo’n twee weken om volledig te herstellen. De laatste jaren zijn er aanwijzingen gekomen dat door de kijkoperatie kankercellen kunnen worden verspreid via het bloed. Maar of die kankercellen ook daadwerkelijk uitzaaiingen kunnen veroorzaken, is nog niet bekend.

Als u inloot in de MRI groep bestaat de mogelijkheid dat de scan suggereert dat de tumor in de blaasspier is gegroeid terwijl dat eigenlijk niet het geval is. Dit zal gebeuren bij ongeveer 1 op de 20 patiënten (5%) bij wie de tumor niet in de blaasspier is gegroeid. U zult dan een agressievere behandeling, meestal bestaande uit een complete blaasverwijdering, krijgen dan gebruikelijk is. Dit komt even vaak voor bij de standaard kijkoperatie. Als de MRI scan suggereert dat de tumor niet is ingegroeid in de blaasspier terwijl dat eigenlijk wel het geval is, dan ondergaat u alsnog de standaard kijkoperatie en zal hiermee in de meeste gevallen de spier-ingroei alsnog kunnen worden aangetoond. In dit geval is het enige nadeel dat u een extra scan heeft gehad.

De MRI scan zelf brengt een extreem kleine kans met zich mee dat u een bijwerking krijgt op de MRI-contrastvloeistof die geïnjecteerd wordt tijdens de scanprocedure. Daarnaast kan voor sommige patiënten de MRI-procedure ongemakkelijk zijn als ze last hebben van claustrofobie. De meeste mensen hebben hier geen last van.

Naast de MRI scan zal ook een klein stukje van de tumor worden weggenomen voor weefselonderzoek door de patholoog. Met deze biopsie wordt de soort kanker en de kwaadaardigheidsgraad bepaald, maar dus niet de ingroei in de spier. Deze biopsie vindt poliklinisch plaats tijdens een blaasspiegeling. Na de biopsie worden eventuele bloedinkjes dicht gebrand. Hiervan voelt u (nagenoeg) niets. Er is een klein risico dat u nog een of enkele dagen klachten heeft van bloed bij de urine.

1. **Wat gebeurt er als u niet wenst deel te nemen aan dit onderzoek?**

U beslist zelf of u meedoet aan het onderzoek. Deelname is geheel vrijwillig. Als u besluit niet mee te doen, hoeft u verder niets te doen. U hoeft niets te tekenen. U hoeft ook niet te zeggen waarom u niet wilt meedoen. U krijgt gewoon de diagnostiek en behandeling die u anders ook zou krijgen, te beginnen met de kijkoperatie met de geheel of gedeeltelijke verwijdering van de tumor. Als u wel meedoet, kunt u zich altijd bedenken en toch stoppen, ook tijdens het onderzoek.

1. **Wat gebeurt er als het onderzoek is afgelopen?**

Het zal ongeveer 3 jaar duren om alle 360 deelnemers te werven voor het onderzoek. Elke deelnemer in het onderzoek zal 2 jaar lang worden gevolgd om te kijken of de ziekte na definitieve behandeling terugkomt. Deze controles zijn hetzelfde als wanneer u niet met het onderzoek mee zou doen. Inclusief de voorbereidingsperiode en het bestuderen en rapporteren van de resultaten zal het onderzoek in totaal 6 jaar duren. Na afloop van dit onderzoek kunt u – indien u dat wenst- een overzicht van de resultaten krijgen.

1. **Bent u verzekerd wanneer u aan het onderzoek meedoet?**

Voor iedereen die meedoet aan dit onderzoek is een verzekering afgesloten. De verzekering betaalt voor schade door het onderzoek. Maar niet voor alle schade. In bijlage B vindt u meer informatie over de verzekering en de uitzonderingen. Daar staat ook aan wie u schade kunt melden.

1. **Wat gebeurt er met uw gegevens?**

Wij laten uw huisarts schriftelijk weten dat u meedoet aan het onderzoek. Tot uw persoon herleidbare onderzoeksgegevens kunnen slechts met uw toestemming door bevoegde personen worden ingezien. Deze personen zijn medewerkers van het onderzoeksteam van uw ziekenhuis, het coördinerend team van het Radboudumc (zij sturen de vragenlijsten naar u en hebben dus inzage in uw persoonsgegevens), radiologen van het Radboudumc die de MRI onderzoeken van andere ziekenhuizen herbeoordelen, medewerkers van de Inspectie voor de Gezondheidszorg, de studiemonitor en leden van de ethische commissie CMO regio Arnhem-Nijmegen. Inzage kan nodig zijn om de betrouwbaarheid en de kwaliteit van het onderzoek na te gaan.

Om uw privacy te beschermen geven wij uw gegevens een code. De sleutel van de code wordt in een beveiligde omgeving bewaard in het ziekenhuis. Als we uw gegevens verwerken, gebruiken we alleen die code. Ook in rapporten en publicaties over het onderzoek kan niemand terughalen dat het over u ging.

We zijn verplicht uw onderzoeksgegevens 15 jaar te bewaren. Daarvoor geeft u toestemming als u meedoet aan dit onderzoek. Als u dat niet wil, kunt u niet meedoen aan dit onderzoek.

We bewaren de volgende gegevens:

- uw naam

- uw geslacht

- uw adres

- uw geboortedatum

- gegevens over uw gezondheid

- (medische) gegevens die we tijdens het onderzoek verzamelen.

De MRI-beelden kunnen van belang zijn voor toekomstig onderzoek. U kunt op de toestemmingsverklaring aangeven of uw het goed vindt dat uw beelden hiervoor gebruikt mogen worden. Uiteraard worden de MRI-beelden altijd alleen onder code gebruikt. Bij het toekomstig gebruik is er een kleine kans dat er op de beelden iets nieuws gevonden wordt dat van belang zou kunnen zijn voor uw behandeling en gezondheid. In dat geval wordt er contact met u opgenomen.

1. **Zijn er extra kosten/is er een vergoeding voor deelname?**

Aan deelname aan de studie zijn voor u geen kosten verbonden. Er is ook geen vergoeding verbonden aan het onderzoek, omdat er voor u in principe geen extra bezoeken aan het ziekenhuis noodzakelijk zijn als u deelneemt. Als u loot voor de kijkoperatie dan moet u daarvoor zoals gebruikelijk naar het ziekenhuis komen. Als u loot voor de MRI groep moet u daarvoor ook naar het ziekenhuis komen, dit ziekenhuisbezoek vervangt dan het bezoek voor de kijkoperatie. U hoeft dan niet opgenomen te worden voor de kijkoperatie. De enige uitzondering hierop is wanneer bij de MRI blijkt dat bij u de tumor toch niet is ingegroeid in de spierlaag. U krijgt dan alsnog een kijkoperatie, maar dan als definitieve behandeling, eventueel gevolgd door een of meerdere blaasspoelingen. Mogelijk beschikt uw ziekenhuis niet over de juiste MRI scanner, in dat geval wordt u doorverwezen naar een ziekenhuis in de buurt. U hoeft niet voor de MRI scan te betalen.

1. **Welke Medisch Ethische Toetsingscommissie heeft dit onderzoek goedgekeurd?**

Dit onderzoek valt onder de Wet medisch-wetenschappelijk onderzoek (WMO) met mensen. De Medisch Ethische Toetsingscommissie (METC) Oost-Nederland heeft het onderzoek goedgekeurd.

1. **Heeft u nog vragen?**

Als u na deze uitleg nog vragen over het onderzoek heeft kunt u die stellen aan de onderzoeker of uw behandelend arts of verpleegkundige.

Wilt u advies van iemand die er geen belang bij heeft? Neemt u dan contact op met Dr. F.M.J. Martens. Hij weet veel over het onderzoek, maar werkt niet mee aan dit onderzoek. U kunt ook contact zoeken met een vertegenwoordiger van de patiëntenvereniging Leven met Blaas of Nierkanker: de heer G. Venderbosch of mevrouw M. Arts.

Heeft u een klacht? Bespreek dit dan met de onderzoeker of de arts die u behandelt. Wilt u dit liever niet? Ga dan naar de onafhankelijke klachtencommissie van het [ziekenhuis]. In bijlage A staat waar u die kunt vinden.

Op de volgende website(s) vindt u desgewenst meer informatie over het onderzoek: https://www.radboudumc.nl/lopende-onderzoeken/BladParadigm. Na het onderzoek zal de website een samenvatting van de resultaten van dit onderzoek tonen. U vindt het onderzoek door te zoeken op ‘BladParadigm’ of ‘Blaaskanker Paradigma’.

1. **Hoe geeft u toestemming voor het onderzoek?**

U kunt eerst rustig nadenken over dit onderzoek. Daarna vertelt u de arts of verpleegkundige die u uit heeft genodigd voor het onderzoek of u de informatie begrijpt en of u wel of niet wilt meedoen. Wilt u meedoen? Dan vult u het toestemmingsformulier in dat u bij deze informatiebrief vindt (bijlage C). U en uw behandelend arts krijgen allebei een getekende versie van deze toestemmingsverklaring.

Dank voor uw tijd.

**Bijlagen bij deze informatie**

A. Contactgegevens

B. Informatie over de verzekering

C. Toestemmingsformulier(en)

**Bijlage A: Contactgegevens**

Als u vragen heeft over het onderzoek of over uw rechten als onderzoeksdeelnemer, kunt u contact opnemen met de hoofdonderzoeker Dr. [naam], [functie], op telefoonnummer […] (secretariaat) of [dienstdoende arts/polikliniek/researchverleegkundige] [naam/namen] op telefoonnummer […].

Voor meer informatie en/of advies over deelname aan het onderzoek kunt u ook contact opnemen met een onafhankelijke arts, dr. F.M.J. Martens op telefoonnummer 024-3613735. Hij is niet betrokken bij het onderzoek, maar is een deskundige op het vlak van het onderzoek.

Als u liever met een vertegenwoordiger van de patiëntenvereniging Leven met Blaas of Nierkanker spreekt kunt u contact zoeken met dhr. G. Venderbosch op telefoonnummer 06-24404772, of met mevr. M. Arts op telefoonnummer 06-38429538.

Als u niet tevreden bent over het onderzoek of de behandeling, dan kunt u contact opnemen met de Functionaris voor de Gegevensbescherming of de Klachtenbemiddelaar.

**Contactgegevens Functionaris voor de Gegevensbescherming**

[Ziekenhuis]

t.a.v. Functionaris Gegevensbescherming

Emailadres: […]

Telefoonnummer: […]

**Contactgegevens Klachtenbemiddeling**

*Let op: niet voor medische klachten/bijwerkingen, bel daarvoor de onderzoeker!*

[Ziekenhuis]

T.a.v. Klachtenfunctionaris

[Antwoordnummer …]

[Postcode + Plaatsnaam]

Emailadres/website: […]

Telefoonnummer: […]

**Bijlage B - Informatie over de verzekering**

Voor iedereen die meedoet aan het onderzoek is een verzekering afgesloten. De verzekering betaalt de schade die u heeft doordat u aan het onderzoek meedeed. Het gaat om schade die u krijgt tijdens het onderzoek, of binnen 4 jaar na het einde van uw deelname aan het onderzoek. U moet schade binnen 4 jaar melden bij de verzekeraar.

Heeft u schade door het onderzoek? Meld dit dan bij deze verzekeraar:

De verzekeraar van het onderzoek is:

Naam verzekeraar: Centramed B.A.

Adres : Postbus 7374, 2701 AJ ZOETERMEER

Telefoonnummer : 070-3017070

E-mail : schade@centramed.nl

Polisnummer : 624.100.021

De verzekering biedt een dekking van € 650.000 per proefpersoon met een maximum van € 5.000.000 voor het hele onderzoek en € 7.500.000 voor schade ten gevolge van medisch-wetenschappelijk onderzoek die per verzekeringsjaar wordt gemeld.

Let op: de verzekering dekt de volgende schade **niet**:

- Schade door een risico waarover we u informatie hebben gegeven in deze brief. Maar dit geldt niet als het risico groter bleek te zijn dan we van tevoren dachten. Of als het risico heel onwaarschijnlijk was.
- Schade aan uw gezondheid die ook zou zijn ontstaan als u niet aan het onderzoek had meegedaan.
- Schade die ontstaat doordat u aanwijzingen of instructies niet of niet goed opvolgde.
- Schade aan de gezondheid van uw kinderen of kleinkinderen.
- Schade door een behandelmethode die al bestaat. Of door onderzoek naar een behandelmethode die al bestaat.

Deze bepalingen staan in het 'Besluit verplichte verzekering bij medisch-wetenschappelijk onderzoek met mensen 2015'. Dit besluit staat in de Wettenbank van de overheid (<https://wetten.overheid.nl>).

**Bijlage C - Toestemmingsformulier proefpersoon**

**BladParadigm studie**

Multiparametrische MRI bij patiënten met verdenking op spier-invasieve blaaskanker: een nieuwe methode om het stadium van de tumor vast te stellen

- Ik heb de informatiebrief gelezen. Ook kon ik vragen stellen. Mijn vragen zijn goed beantwoord. Ik had genoeg tijd om te beslissen of ik meedoe.
- Ik weet dat meedoen vrijwillig is. Ook weet ik dat ik op ieder moment kan beslissen om ermee te stoppen. Ik hoef dan niet te zeggen waarom ik wil stoppen.
- Ik geef toestemming om gegevens uit het medisch dossier op te vragen bij de uroloog die mij behandelt en bij reeds bestaande landelijke bevolkings- en ziekteregisters (o.a. de kankerregistratie van IKNL, de pathologieregistratie van PALGA, en de Basis Registratie Personen). Het gaat hier alleen om de gegevens die van belang zijn voor dit onderzoek.
- Ik geef tevens toestemming om de gegevens die verzameld zijn binnen dit onderzoek te gebruiken voor andere wetenschappelijke vraagstellingen, mits de ethische commissie daarvoor toestemming geeft.
- Ik geef

wel

geen

toestemming voor toekomstig nader gebruik van de MRI-beelden.

- Ik weet dat sommige mensen mijn medische gegevens kunnen inzien. Die mensen staan vermeld in deze informatiebrief.
- Ik wil meedoen aan dit onderzoek.

Naam: ……………………………….. Geboortedatum: __ / __ / __

Handtekening: ……………………… Datum: __ / __ / __

----------------------------------------------------------------------------------------------------------------------------

Ik verklaar dat ik deze proefpersoon volledig heb geïnformeerd over het genoemde onderzoek.

Wordt er tijdens het onderzoek informatie bekend die de toestemming van de proefpersoon kan beïnvloeden? Dan laat ik dit op tijd weten aan deze proefpersoon.

Naam onderzoeker / behandelend arts (of diens vertegenwoordiger):

……………………………………….

Handtekening:……………………… Datum: __ / __ / __

-----------------------------------------------------------------------------------------------------------------

*De proefpersoon krijgt een volledige informatiebrief mee, samen met een getekende versie van het toestemmingsformulier*
