# Supplementary material for: Multiparametric MRI for local staging in patients with suspected muscle-invasive bladder cancer: study protocol for a multicentre, non-inferiority randomised controlled trial (the BladParadigm study)
Source: BMJ Open. 2025 Aug 16;15(8):e100002. doi: 10.1136/bmjopen-2025-100002 (PMC12359491; doi:10.1136/bmjopen-2025-100002)
Supplement: Supplementary file 2 [file bmjopen-2025-100002.reviewer_comments.pdf]

PEER REVIEW HISTORY

BMJ Open publishes all reviews undertaken for accepted manuscripts. Reviewers are asked to complete a checklist review form and are provided with free text boxes to elaborate on their assessment. These free text comments are reproduced below.

ARTICLE DETAILS

Title (Provisional)

Multiparametric MRI for local staging in patients with suspected muscle-invasive bladder cancer: study protocol for a multicenter, non-inferiority randomized controlled trial (the BladParadigm study)

Authors

van Koeverden, Sebastiaan; van Hoogstraten, Lisa; de Rooij, Maarten; van der Leest, Marloes; Grutters, Janneke; Appendix, BladParadigm Study Group; Kiemeney, Lambertus A.; van der Heijden , Toine

---

VERSION 1 - REVIEW

**Reviewer**	**1**
**Name**	**Arita, Yuki**
**Affiliation**	**Memorial Sloan Kettering Cancer Center, Radiology**
**Date**	**06-Mar-2025**
**COI**	**None**

Summary of the Review

Study Design and Manuscript Contents

・The manuscript is based on a good randomized controlled trial (RCT) which compared an mpMRI based diagnostic approach with standard TURBT in patients with suspected MIBC.

・The study design is good and clinically meaningful in order to explain an significant diagnostic and oncologic dilemma in bladder cancer management.

・The methodology is quite extensive and dates back to rather sophisticated studies, involving imaging protocols, biopsy techniques, and cost analysis.

・The primary and secondary endpoints are clearly defined, especially the progression free survival (PFS) at two years.

Major Strengths

• New diagnostic paradigm: This study is one of the first large-scale, multicenter RCTs assessing the role of mpMRI in the staging of suspected MIBC patients.

• Direct clinical impact: The study aims to enhance the oncologic results by preventing the potential risks of tumor seeding and the delays associated with TURBT.

• Robust methodology: The study design guarantees the generalization across the centers, the imaging review and the mpMRI acquisition with VI-RADS.

• Patient centered approach: The study also includes patient reported outcomes (QoL assessments) and cost effectiveness analysis.

Major Weaknesses

• Possible heterogeneity in mpMRI interpretation: Despite the efforts to unify the reporting through the training in VI-RADS, the inter reader variability is still an issue.

• Inclusion bias: The study includes only patients with high cystoscopic suspicion (Likert score 4-5) which may affect the generalization of the results.

• Risk of overtreatment: The classification of VI RADS 3 as muscle invasive may increase the rates of radical treatment in some non MIBC cases.

• Low estimation of the risk of muscular invasion: Patients with initial mpMRI diagnosis of non MIBC who turned out to have MIBC may experience a delay in receiving appropriate treatment.

Detailed Comments

Title

Strength: The title is relevant and explains what the study aims to accomplish with regards to mpMRI-based staging of MIBC.

Weakness: It may help to explicitly state "randomized controlled trial" in the title to emphasize the study type.

Suggested Revision:

"Multi-parametric MRI for Local Staging of Muscle Invasive Bladder Cancer: A Randomized Controlled Trial (The BladParadigm Study)."

Abstract

Strengths:

• Provides clear statement of the problem, methods, results, and conclusions.

• Includes appropriate terms for indexing and retrieval.

• Primary and secondary endpoints are presented well.

Weaknesses:

• The discussion could be more concise and the focus could be made on the major consequences instead of repeating the methods.

• It may be useful to include some numerical values (for instance, the calculated power, the expected PFS gain) to make the study more compelling.

Suggested Revisions:

• The phrase “oncologically safer, faster, and less costly” could be more precise, e.g.,

“A potentially safer and even more economical approach for local staging in bladder cancer.”

1. Introduction

Strengths:

• Why mpMRI should be investigated as an alternative to TURBT is well explained.

• Presents the drawbacks of TURBT, including the risk of tumor spread and delayed diagnosis.

• Supports the findings with appropriate references and previous meta-analysis of mpMRI accuracy.

Weaknesses:

• The hypothesis statement can be more concise.

• Does not have a specific reference to the earlier studies (e.g. BladderPath) that helped in the design of this trial.

Suggested Revisions:

• The current review of the background information on the risks of TURBT can be reduced to one paragraph.

• A sentence should be included that clearly relates the findings of BladderPath to the reason for this trial.

2. Materials and Methods

Strengths:

• The randomization process and the imaging central review are well explained.

• The use of training of radiologists in VI RADS is a significant advantage because of the diagnostic consistency.

• The patient selection criteria and the study workflow are well described.

Weaknesses:

• The management of the VI RADS 3 lesions could have been explained in more detail.

• There is no mention of how within reader agreement will be addressed.

• There is no discussion of the plans for sensitivity analysis for missing data in the statistical analysis plan.

3. Discussion

Strengths:

• Presents the clinical relevance and the possibility of changing the current approach to bladder cancer diagnosis.

• Presents considerations of economic efficiency.

• Suggests possible drawbacks, such as the variability of the diagnosis made by VI RADS.

• The BladderPath study is not fully compared with.

• The VI RADS 3 risk of overtreatment requires further analysis.

• The long term oncological results of the study are not presented, including the period beyond two years.

Suggested Revisions:

• More details should be provided on how BladderPath informed the expectations of the study.

• How to avoid over treatment in patients with VI RADS 3.

• Discuss the need for long term follow up beyond two years.

4. Conclusion

Strengths:

• Summarizes the study goals and what might be gained from the study.

• Restates the possible advantages of mpMRI compared to TURBT.

Weaknesses:

• It could be more specific on how the findings will influence practice.

• It may be useful to mention some possible directions (for example, guideline recommendation, other similar studies).

5. References

Strengths: Covers all possible sources and includes a large number of appropriate references.

References the major guidelines and meta analyses.

Reviewer **2**

Name **Clarke, Caroline**

Affiliation **University College London, Research Department of Primary Care and Population Health**

Date **23-May-2025**

COI **None**

Thank you for the opportunity to review this very clearly written paper.

I am a health economist working primarily on economic evaluations alongside trials so have focused on these aspects.

Abstract

Could mention geography/region of the study

Figure 1

I got slightly lost here – why is TURBT in there twice on the right-hand side?

Page 11, Central radiology review

Double readings – does this refer to readings of the MRI? Would be helpful to specify here.

Page 13

Could please add citation (with url if at all possible) for the Dutch manual

Page 15

Is a 2-year time horizon enough for the cost-effectiveness analysis, or should it be lifetime?

Further to this point: the study flowchart in Fig 1 shows what will happen to the patients, which makes sense for the running of the trial, but the cost-effectiveness analysis might need to also consider false positives / false negatives in case there are impacts from these further down the line – or are the MIBC and NMIBC considered to be 100% accurate?

General comments

What will the recruitment rate be like? If I saw this paper as a patient then I would refuse to have the TURBT at all, and if it was the only standard of care option then it would make me very nervous of having it. The authors do not seem to have equipoise, so it might be hard to recruit patients to be randomised across these two arms.

In terms of implementation of the new procedure (mpMRI + same day biopsy), are there any implementation considerations that need to be looked at, e.g. in a process evaluation? If the study result is positive, can this be rolled out nationwide, or would there be any staff capacity / timings / booking systems / logistical etc. barriers to implementing the new procedure?

VERSION 1 - AUTHOR RESPONSE

Reviewer 1 Comments to the Author:

• The manuscript is based on a good randomized controlled trial (RCT) which compared an mpMRI based diagnostic approach with standard TURBT in patients with suspected MIBC
• The study design is good and clinically meaningful in order to explain an significant diagnostic and oncologic dilemma in bladder cancer management.
• The methodology is quite extensive and dates back to rather sophisticated studies, involving imaging protocols, biopsy techniques, and cost analysis.
• The primary and secondary endpoints are clearly defined, especially the progression free survival (PFS) at two years.

Major Strengths

• New diagnostic paradigm: This study is one of the first large-scale, multicenter RCTs assessing the role of mpMRI in the staging of suspected MIBC patients.
• Direct clinical impact: The study aims to enhance the oncologic results by preventing the potential risks of tumor seeding and the delays associated with TURBT.
• Robust methodology: The study design guarantees the generalization across the centers, the imaging review and the mpMRI acquisition with VI-RADS.
• Patient centered approach: The study also includes patient reported outcomes (QoL assessments) and cost effectiveness analysis.

Major Weaknesses

• Possible heterogeneity in mpMRI interpretation: Despite the efforts to unify the reporting through the training in VI-RADS, the inter reader variability is still an issue.
• Inclusion bias: The study includes only patients with high cystoscopic suspicion (Likert score 4-5) which may affect the generalization of the results.
• Risk of overtreatment: The classification of VI RADS 3 as muscle invasive may increase the rates of radical treatment in some non MIBC cases.
• Low estimation of the risk of muscular invasion: Patients with initial mpMRI diagnosis of non MIBC who turned out to have MIBC may experience a delay in receiving appropriate treatment.

We thank the reviewer for the detailed review. We are happy to read that our study protocol was well-described in the manuscript and that the reviewer agrees with the strengths of the study. We are aware of the limitations of our study and have addressed them mainly in the discussion section (page 24-25). A more detailed response is provided below.

Detailed Comments

Title
Strength: The title is relevant and explains what the study aims to accomplish with regards to mpMRI-based staging of MIBC.
Weakness: It may help to explicitly state "randomized controlled trial" in the title to emphasize the study type.

Suggested Revision:
"Multi-parametric MRI for Local Staging of Muscle Invasive Bladder Cancer: A Randomized Controlled Trial (The BladParadigm Study)."

We have adapted the title as follows, taking the comments from the editor into account as well:

Multiparametric MRI for local staging in patients with suspected muscle-invasive bladder cancer: study protocol for a multicenter, non-inferiority randomized controlled trial (the BladParadigm study)

Abstract
Strengths:
• Provides clear statement of the problem, methods, results, and conclusions.
• Includes appropriate terms for indexing and retrieval.
• Primary and secondary endpoints are presented well.

Weaknesses:
• The discussion could be more concise and the focus could be made on the major consequences instead of repeating the methods.

As suggested by the editor, we have removed the discussion section from the abstract, as it does not comply with the BMJ format. Instead, we have expanded the Strengths and limitations section below the abstract (page 4-5):

"Strengths and limitations of this study

- This is one of the first, large, multicenter, randomized controlled trials comparing an mpMRI-based diagnostic pathway to current standard of care, both in academic and community hospitals.
- All mpMRI scans follow a standardized VI-RADS-based protocol for acquisition and reporting.
- All mpMRI scans will be double-read to ensure robust diagnosis.
- At the end of the study, TURBT and radical cystectomy specimens of the intervention arm will undergo central review to assess the diagnostic validity of mpMRI.
- Inclusion is based on cystoscopic assessment, which is subjective and may vary between clinicians."

• It may be useful to include some numerical values (for instance, the calculated power, the expected PFS gain) to make the study more compelling.

We have added the following sentence to the abstract (page 3):

"The study powered to demonstrate that the mpMRI-based strategy is at least non-inferior to standard TURBT in patients treated with radical cystectomy alone, assuming a relative hazard of 0.55. The required sample size is 360 patients (180 TURBT, 180 mpMRI)."

Suggested Revisions:
• The phrase "oncologically safer, faster, and less costly" could be more precise, e.g., "A potentially safer and even more economical approach for local staging in bladder cancer."

We have slightly adapted this sentence (page 3):

"The aim of this randomized study is to determine whether multiparametric MRI (mpMRI) of the bladder, in combination with a single outpatient bladder tumor biopsy for histological confirmation,

is a safer, faster, less costly, and therefore more cost-effective diagnostic pathway than TURBT to detect or rule out MIBC."

We have chosen not to change the outcomes into 'more economical', as the outcomes as they are stated now, directly corresponds to our primary and secondary outcome measures. We have, however, added a sentence in the discussion section of the main text, with suggested changes (page 26):

"Notwithstanding these limitations, if the results of this RCT show that this new diagnostic approach combining mpMRI with a same-day bladder biopsy is a reliable, safer, faster and more economical alternative for local staging of bladder cancer compared to TURBT, it will pave the way for a paradigm shift in bladder cancer staging."

1. Introduction
Strengths:
- **Why mpMRI should be investigated as an alternative to TURBT is well explained.**
- **Presents the drawbacks of TURBT, including the risk of tumor spread and delayed diagnosis.**
- **Supports the findings with appropriate references and previous meta-analysis of mpMRI accuracy.**

Weaknesses:
- **The hypothesis statement can be more concise.**
- **Does not have a specific reference to the earlier studies (e.g. BladderPath) that helped in the design of this trial.**

Suggested Revisions:
- **The current review of the background information on the risks of TURBT can be reduced to one paragraph.**

We have revised the section accordingly and summarised the background on TURBT risks into one concise paragraph (page 6):

"Transurethral resection of the bladder tumour (TURBT) is the current standard for both pathological confirmation and local staging of bladder cancer, but it has several limitations. Unlike most cancers, which are staged non-invasively through imaging, bladder cancer requires TURBT, an invasive procedure that involves cutting through the tumor and its surrounding vasculature, potentially leading to tumor cell dissemination. Small studies have demonstrated the presence of circulating tumour cells (CTCs) in the blood following TURBT, supporting the hypothesis of procedure-induced tumour cell spread (2-4). Additionally, TURBT may underestimate muscle invasion due to inadequate sampling and tissue handling, necessitating re-TURBT in up to 30% of cases (5-8). These limitations may delay definitive treatment and contribute to the persistently poor prognosis of muscle-invasive bladder cancer (MIBC), despite advances in curative therapies (9)."

- **A sentence should be included that clearly relates the findings of BladderPath to the reason for this trial.**

We have incorporated the BladderPath trial into the rationale for conducting this trial (page 7):

"A recent UK-based trial (BladderPath) (13, 14) introduced an MRI-directed diagnostic pathway for patients with suspected MIBC and showed that it is feasible to incorporate mpMRI into the diagnostic pathway for patients with bladder cancer, and this led to a significant reduction in time to

correct treatment. ; hHowever, since oncological outcomes such as progression-free survival weare not evaluated still pending, ourthe BladParadigm trial is designed to evaluate whether an mpMRI-based approach can safely replace TURBT in terms of both effectiveness and costs.
”

2. Materials and Methods
Strengths:
• The randomization process and the imaging central review are well explained.
• The use of training of radiologists in VI RADS is a significant advantage because of the diagnostic consistency.
• The patient selection criteria and the study workflow are well described.

Weaknesses:
• The management of the VI RADS 3 lesions could have been explained in more detail.

We have elaborated on this in more detail on page 25-26:

“Another methodological consideration is the use of VI-RADS 3 as a threshold for classifying patients as MIBC. In the original VI-RADS paper, a score of 3 is considered equivocal In addition, it is somewhat arbitrary to consider VI-RADS 3 as MIBC. In the original VI-RADS paper, a score of 3 is considered equivocal for MIBC (16). While more recent studies report high diagnostic performance for VI-RADS ≥3 — with sensitivities of 87–91% and specificities of 82–97% More recent studies show an excellent sensitivity (87-91%) and specificity (82-97%) when using a cut-off score of 3 and higher for the presence of MIBC (12, 28, 29) — the risk of overtreatment in non–muscle-invasive cases cannot be entirely excluded.. A meta-analysis by Woo et al. (n=1170) found a sensitivity and specificity of 92% and 84% for VI-RADS 3–5, compared to 72% and 96% for VI-RADS 4–5, respectively. Although a higher cut-off would reduce false positives, we chose to include VI-RADS 3 in the MIBC category for two reasons. First, the pre-test probability of MIBC in our study population is high (72–78%) because patients are only included if cystoscopy suggests a high likelihood of muscle invasion (Likert score 4 or 5). In this context, the positive predictive value of VI-RADS 3–5 rises to 93.6–95.4%. Second, although inter-reader variability in mpMRI interpretation remains an inherent limitation, the use of central double reading with consensus reporting is expected to improve consistency and reduce the number of equivocal (VI-RADS 3) classifications.

We acknowledge that overstaging may lead to overtreatment, particularly in patients with large, aggressive-looking tumors that may still be non–muscle-invasive. In clinical practice, however, such tumors are often treated more aggressively regardless of histological confirmation, and radical cystectomy is not uncommon in these cases. Although there is no direct evidence quantifying overtreatment in this context, we estimate that its occurrence is low—likely below 5%—corresponding to a positive predictive value above 95%. Conversely, if mpMRI understages an MIBC case and incorrectly classifies it as NMIBC, the patient will still undergo TURBT as per protocol, allowing for detection of muscle invasion at that point. In such cases, the primary consequence could be a delay of several days due to the initial mpMRI and biopsy procedure.”

• There is no mention of how within reader agreement will be addressed.

We have clarified the assessment of the inter-reader variability in the methods section (page 13-14): “the inter-reader variability among radiologists will be assessed by calculating the intraclass correlation coefficient.”

Regarding the within reader agreement, or intra-reader variability, this will not be assessed as all mpMRI scans will be read once per reader and consensus will be reached through central double reading in case of disagreement. This approach was chosen to reflect real-world clinical practice, where radiologists typically assess imaging once rather than multiple times. In addition, health care professionals, including radiologists, are under enormous pressure and have limited time and resources. As the mpMRI-pathway is ultimately to be implemented in current practice, we have chosen minimize the additional burden of this trial. To ensure the quality of the mpMRI images and reporting, we provided an elaborate, obligatory training on the mpMRI protocol and VI-RADS scoring system for all participating radiologists, after first having completed extensive training at the experienced radiology group from Professor Panebianco in Italy (please see page 12).

• There is no discussion of the plans for sensitivity analysis for missing data in the statistical analysis plan.

We have clarified in the statistical analysis section that sensitivity analyses for missing data will be conducted using imputation-based approaches and compared to complete case analyses (page 21):

"Missing data will be handled using multiple imputation with sensitivity analyses comparing imputed and complete case results. Pre-specified subgroup and sensitivity analyses, for instance, stratified by use of neoadjuvant chemotherapy, will be conducted."

3. Discussion
Strengths:
• Presents the clinical relevance and the possibility of changing the current approach to bladder cancer diagnosis.
• Presents considerations of economic efficiency.
• Suggests possible drawbacks, such as the variability of the diagnosis made by VI RADS.

• The BladderPath study is not fully compared with.
• The VI RADS 3 risk of overtreatment requires further analysis.
• The long term oncological results of the study are not presented, including the period beyond two years.

Suggested Revisions:
• More details should be provided on how BladderPath informed the expectations of the study.

We have added an in-depth discussion of the BladderPath trial in the discussion section (page 24):

"To the best of our knowledge, the BladderPath study is the only other trial that is investigated an mpMRI-based diagnostic pathway for bladder cancer staging. Results on feasibility and time to correct therapy were recently published (13, 14). The introduction of an MRI based diagnostic approach led to a 45-day reduction in time to correct therapy. The findings from this study emphasize the importance of staging patients suspected of MIBC with mpMRI rather than TURBT. However, survival data were not evaluated due to insufficient enrollment due to the COVID-19 pandemic. To optimize our study design, we have consulted with the principal investigator of the BladderPath trial and made several adaptations, including the use of a 3T mpMRI (instead of a 1.5T mpMRI as done in the UK-trial), evaluating progression-free survival at 2 years after randomization as our primary endpoint, and a more stringent selection of only patients with a suspicion of MIBC (Likert score 4 or 5 during cystoscopy, instead of all patients with gross hematuria). The latter, however, could also be considered a limitation, as cystoscopic-based assessment of potential

muscle-invasion is subject to variability and potential inaccuracies. This was shown in two studies on accuracy of cystoscopy where the prevalence of MIBC among patients with a Likert score 4 or 5 was respectively 72 and 78% (26, 27)."

• How to avoid over treatment in patients with VI RADS 3.

We have elaborated on potential overtreatment in patients with VI-RADS 3 in the discussion section (page 25-26):

"Another methodological consideration is the use of VI-RADS 3 as a threshold for classifying patients as MIBC. In the original VI-RADS paper, a score of 3 is considered equivocal In addition, it is somewhat arbitrary to consider VI-RADS 3 as MIBC. In the original VI-RADS paper, a score of 3 is considered equivocal for MIBC (16). While more recent studies report high diagnostic performance for VI-RADS ≥3 — with sensitivities of 87–91% and specificities of 82–97% More recent studies show an excellent sensitivity (87-91%) and specificity (82-97%) when using a cut-off score of 3 and higher for the presence of MIBC (12, 28, 29) — the risk of overtreatment in non–muscle-invasive cases cannot be entirely excluded.. A meta-analysis by Woo et al. (n=1170) found a sensitivity and specificity of 92% and 84% for VI-RADS 3–5, compared to 72% and 96% for VI-RADS 4–5, respectively. Although a higher cut-off would reduce false positives, we chose to include VI-RADS 3 in the MIBC category for two reasons. First, the pre-test probability of MIBC in our study population is high (72–78%) because patients are only included if cystoscopy suggests a high likelihood of muscle invasion (Likert score 4 or 5). In this context, the positive predictive value of VI-RADS 3–5 rises to 93.6–95.4%. Second, although inter-reader variability in mpMRI interpretation remains an inherent limitation, the use of central double reading with consensus reporting is expected to improve consistency and reduce the number of equivocal (VI-RADS 3) classifications.

We acknowledge that overstaging may lead to overtreatment, particularly in patients with large, aggressive-looking tumors that may still be non–muscle-invasive. In clinical practice, however, such tumors are often treated more aggressively regardless of histological confirmation, and radical cystectomy is not uncommon in these cases. Although there is no direct evidence quantifying overtreatment in this context, we estimate that its occurrence is low—likely below 5%—corresponding to a positive predictive value above 95%. Conversely, if mpMRI understages an MIBC case and incorrectly classifies it as NMIBC, the patient will still undergo TURBT as per protocol, allowing for detection of muscle invasion at that point. In such cases, the primary consequence could be a delay of several days due to the initial mpMRI and biopsy procedure."

• Discuss the need for long term follow up beyond two years.

We have elaborated on this in the discussion section (page 25):

"Equal to the BladderPath trial, follow-up will be at least two years for all patients. Given that most patients who experience progression, will have progression in the first two years after radical treatment, two years is a sufficiently large time window for follow-up. It would be of added value to extend follow-up in this trial beyond two years to evaluate long-term results, but due to feasibility constraints this was not possible. Patients will, however, be monitored long-term according to standard of care."

4. Conclusion

Strengths:
• Summarizes the study goals and what might be gained from the study.
• Restates the possible advantages of mpMRI compared to TURBT.

Weaknesses:
• It could be more specific on how the findings will influence practice.
• It may be useful to mention some possible directions (for example, guideline recommendation, other similar studies).

As suggested by the editor, we have removed the conclusion section from the main text, as it does not comply with the BMJ format for a study protocol. Instead, we have added a final statement to the discussion section regarding the potential influence on practice (page 26):

"Notwithstanding these limitations, if the results of this RCT show that this new diagnostic approach combining mpMRI with a same-day bladder biopsy is a reliable, safer, faster and more economical alternative for local staging of bladder cancer compared to TURBT, it will pave the way for a paradigm shift in bladder cancer staging."

5. References
Strengths: Covers all possible sources and includes a large number of appropriate references. References the major guidelines and meta analyses.

Based on the comments from reviewer #2 we have added one reference. We have not made any other changes to the reference section.

<u>Reviewer 2 Comments to the Author:</u>
Thank you for the opportunity to review this very clearly written paper.
I am a health economist working primarily on economic evaluations alongside trials so have focused on these aspects.

We thank the reviewer for the detailed review. We are happy to read that our study protocol was clearly written. We have addressed the comments point-per-point below. Please note that the page numbers and headings have changed, as the format of the manuscript was adapted based on suggestions of the editor.

Abstract: Could mention geography/region of the study

We have added a statement about the geography of the study (page 3):

"BladParadigm is a two-arm multicenter randomized controlled trial (RCT) conducted in the Netherlands."

Figure 1: I got slightly lost here – why is TURBT in there twice on the right-hand side?

In case the mpMRI result is VI-RADS 1 or 2, we consider the bladder tumor to be non-muscle invasive. In case of NMIBC, standard of care consists of TURBT. In the figure, this step is described as 'TURBT', but could also have been written down as 'Usual care'. However, since the aim of this study

is to evaluate mpMRI versus TURBT, we have consciously chosen to describe this step separately. Based on the results of TURBT, the definitive diagnosis is made and subsequent treatment is decided on, hence again the distinction between NMIBC and MIBC. We have elaborated on this in the manuscript as well (page 14 and 15).

"Results from the mpMRI and biopsy are discussed in a multidisciplinary tumor board, where definitive treatment plans will be determined. Patients with a VI-RADS score of 1 or 2 will be classified as having NMIBC and will typically undergo TURBT, often supplemented by bladder instillations and/or re-TURBT. Those with a VI-RADS score of 3, 4 or 5 will be classified as having MIBC and will undergo definitive treatment, usually involving radical cystectomy (RC) with or without neoadjuvant chemotherapy (NAC) or chemoradiotherapy."

"It is anticipated that mpMRI will classify a maximum of 10% of patients as NMIBC, who will then undergo TURBT."

Page 11, Central radiology review: Double readings – does this refer to readings of the MRI? Would be helpful to specify here.

We have specified this better (page 13):

"Double-readings of the mpMRI scans will be performed at the main investigator's location (Radboudumc) by a dedicated team of radiologists."

Page 13: Could please add citation (with url if at all possible) for the Dutch manual

We have added a citation for the Dutch manual for costing studies (page 16 and reference section page 34-35):

"Zorginstituut Nederland. Guideline for conducting economic evaluations in healthcare [in Dutch: Richtlijn voor het uitvoeren van economische evaluaties in de gezondheidszorg]. 2024 (https://www.zorginstituutnederland.nl/publicaties/publicatie/2024/01/16/richtlijn-voor-het-uitvoeren-van-economische-evaluaties-in-de-gezondheidszorg). Accessed 20 June 2025."

Page 15: Is a 2-year time horizon enough for the cost-effectiveness analysis, or should it be lifetime?

With this trial, we interfere in the diagnostic pathway. Further treatment and monitoring of the bladder cancer will be based on current (standard) clinical care. As most patients who experience progression, develop progression of the disease within 2 years, and given that the expected gain in time to definitive treatment and quality of life will occur shortly after TURBT/mpMRI, we therefore expect that the effect, i.e., the (difference in) cost-effectiveness, will also occur within this time frame. Therefore, after careful deliberation with a health technology expert involved in the trial, we have set the time horizon at 2 years.

Further to this point: the study flowchart in Fig 1 shows what will happen to the patients, which makes sense for the running of the trial, but the cost-effectiveness analysis might need to also consider false positives / false negatives in case there are impacts from these further down the line – or are the MIBC and NMIBC considered to be 100% accurate?

Diagnosing NMIBC/MIBC is never 100% accurate, with either mpMRI or TURBT. Following the diagnosis, patients will undergo further diagnostic interventions or definitive treatment. All actions following diagnosis, whether true or false, will be registered and costs will be calculated accordingly. We will thus take into account whether a true or false diagnosis was given, in order to give an accurate reflection of the cost-effectiveness.

General comments

What will the recruitment rate be like? If I saw this paper as a patient then I would refuse to have the TURBT at all, and if it was the only standard of care option then it would make me very nervous of having it. The authors do not seem to have equipoise, so it might be hard to recruit patients to be randomised across these two arms.

We completely agree with the reviewer. This lack of equipoise was actually the reason to initiate this trial. For decades, there have not been suitable alternatives for TURBT. Since its invention in 1910, TURBT has been standard of care. We have described the risks and benefits of TURBT and mpMRI (+ biopsy) extensively in the patient information folder, after consulting both the medical ethical committee as well as patient representatives from the Dutch patient association. The current standard of care is TURBT, so without participation in the trial, they will receive this anyway. When participating, there is a 50% chance of receiving mpMRI plus a same-day biopsy. The trial is currently recruiting, and we observe that patients are actually very willing to participate.

In terms of implementation of the new procedure (mpMRI + same day biopsy), are there any implementation considerations that need to be looked at, e.g. in a process evaluation? If the study result is positive, can this be rolled out nationwide, or would there be any staff capacity / timings / booking systems / logistical etc. barriers to implementing the new procedure?

If the result is positive, the next step would be to disseminate our results and incorporate them in the clinical guidelines. Then, hospitals can start implementing mpMRI into their diagnostic pathway (both in the Netherlands and in Europe). The trial is currently recruiting in 14 hospitals in the Netherlands, so we already have a lot of experience with implementing mpMRI in clinical practice (albeit in a study-setting). We experienced quite some differences between hospitals, and have deliberated carefully with each local study site on how to proceed. For instance, not all hospitals have a 3T mpMRI scanner and therefore need to refer patients to other hospitals. The hospitals that do have a 3T mpMRI scanner, are becoming experts in its use for bladder cancer diagnosis, and it is very likely that when mpMRI will be implemented nationally, these hospitals will be expert centers who can receive referrals from nearby hospitals. This will also positively influence the quality of the mpMRI scans and the patients' diagnosis.

For this study, we have consulted an implementation expert. Together, we will perform a qualitative study on barriers and facilitators among focus groups of patients, urologists and radiologists, and use this, next to the experience of running the trial, as input for a tailored implementation strategy and the development of further relevant products.

VERSION 2 - REVIEW

**Reviewer**	**1**
**Name**	**Arita, Yuki**
**Affiliation**	**Memorial Sloan Kettering Cancer Center, Radiology**
**Date**	**19-Jul-2025**
**COI**	

The manuscript is also well organized and clearly written. I have no major concerns, and I believe it is suitable for publication in its current form.
